# Deep learning reveals diverging effects of altitude on aging

**DOI:** 10.1007/s11357-024-01502-8

**Published:** 2025-01-15

**Authors:** Amanuel Abraha Teklu, Indra Heckenbach, Michael Angelo Petr, Daniela Bakula, Guido Keijzers, Morten Scheibye-Knudsen

**Affiliations:** 1 Center for Healthy Aging, Department of Cellular and Molecular Medicine, University of Copenhagen, Copenhagen, Denmark; 2 Department of Biochemistry and Molecular Biology, College of Health Sciences, Mekelle University, Mekelle, Ethiopia; 3 Tracked Biotechnologies, LLC, Manassas, VA USA

**Keywords:** Risk exposure data, Disease burden data, High altitude, Aging, Senescence, Photoaging, Risk factors, Disease burden

## Abstract

Aging is influenced by a complex interplay of multifarious factors, including an individual’s genetics, environment, and lifestyle. Notably, high altitude may impact aging and age-related diseases through exposures such as hypoxia and ultraviolet (UV) radiation. To investigate this, we mined risk exposure data (summary exposure value), disease burden data (disability-adjusted life years (DALYs)), and death rates and life expectancy from the Global Health Data Exchange (GHDx) and National Data Management Center for Health of Ethiopia for each subnational region of Ethiopia, a country with considerable differences in the living altitude. We conducted a cross-sectional clinical trial involving 227 highland and 202 lowland dwellers from the Tigray region in Northern Ethiopia to gain a general insight into the biological aging at high altitudes. Notably, we observed significantly lower risk exposure rates and a reduced disease burden as well as increased life expectancy by lower mortality rates in higher-altitude regions of Ethiopia. When assessing biological aging using facial photographs, we found a faster rate of aging with increasing elevation, likely due to greater UV exposure. Conversely, analysis of nuclear morphologies of peripheral blood mononuclear cells (PBMCs) in blood smears with five different senescence predictors revealed a significant decrease in DNA damage-induced senescence in both monocytes and lymphocytes with increasing elevation. Overall, our findings suggest that disease and DNA damage-induced senescence decreases with altitude in agreement with the idea that oxidative stress may drive aging.

**Supplementary Information:**

The online version contains supplementary material available at 10.1007/s11357-024-01502-8.

## Introduction

Aging and age-related diseases are critically regulated by multifarious factors pertaining to the genetics, metabolism, behavior, lifestyle, and environment of the individual or population [1]. The role of oxidative stress in aging, particularly how reduced or increased oxygen exposure affects aging, remains a debated topic [2]. At high altitudes, hypoxia occurs due to a decrease in the partial pressure of oxygen in the air, accompanied by increased exposure to ultraviolet (UV) radiation [3]. While oxygen is essential for cellular energy production in the form of ATP through oxidative phosphorylation in the mitochondria, it is also highly reactive and can oxidize proteins, lipids, DNA, and other macromolecules in cells. Data suggests that cells incubated under hypoxic conditions may exhibit increased genetic instability [4], altered metabolism [5], and cell death activities [6] as well as therapy-resistant behaviors [7]. Nevertheless, there are emerging studies demonstrating the beneficial role of hypoxia in health and aging. For instance, culturing primary cells in low oxygen can improve cell survival [8] and hypoxia is required for maintaining the stemness of stem cells [9]. Further, reduced oxygen levels have been implicated in the considerable longevity of naked mole rats which reside in hypoxic underground tunnels and live longer, healthier lives compared to similarly sized rodents in normoxic environments [10]. In addition, nematodes exposed to moderate hypoxia have shown extended lifespan by up to 40 percent [11].

However, there are currently no aging studies on native human population living at high-altitude hypoxia to validate these findings. Investigating the impact of hypoxia on human aging is challenging due to the limited number of populations living at both high and low altitudes. Therefore, Ethiopia is of particular interest because it contains the highest proportion of population in the world permanently living at high altitude (> 1500 masl) [12]. At the organismal level, the body undergoes adaptation at high altitude, where tissues consume reduced oxygen by entering into a hypometabolic state to adjust to the low level of oxygen at high altitudes [13]. In addition, recent studies have also identified nitric oxide (NO) and cyclic guanosine monophosphate (cGMP)–mediated vasodilation as a mechanism of adaptation to high altitude chronic hypoxia [14]. Although organisms employ various adaptative mechanisms to hypoxia and other stimuli to maintain homeostasis, emerging evidence suggests these mechanisms decline with age [15]. For example, aged mice show a reduced HIF1 function compared to younger mice [16]. In addition, in a study conducted on aged human diploid fibroblasts, a significant reduction in HIF1 binding efficiency to the sequences of hypoxia response elements (HREs) was observed [17]. Furthermore, as age advances, oxygen sensing [18] and the ventilatory system of the body [19] also changes, potentially leading to a relative hypoxia. Weakening of the respiratory system combined with the failure of the adaptation machinery to hypoxia with age may increase the vulnerability of older individuals, especially those dwelling at higher altitudes.

Taken together, existing in vitro, data, animal models, and human studies suggest that high altitude may influence human aging [10, 17, 20, 21]. To test this hypothesis, we initially assessed the risk factors and disease burden and their influence on healthspan and lifespan in lowland and highland regions of Ethiopia using summary exposure value (SEV), disability-adjusted life years (DALYs), death rates and life expectancy data from the Global Health Data Exchange (GHDx) and National Data Management Center for Health of Ethiopia, respectively. We then cross-sectionally collected biomarkers (facial images and nuclear images of immune cells from peripheral blood) from a cohort of 429 human volunteers dwelling at varying altitudes (highlands and lowlands) in the Tigray region of Northern Ethiopia. Using computational approaches, we examined whether these biomarkers of biological aging were altered in response to altitude. Overall, this study provides new insights into the rate of biological aging and the levels of risk factors and disease burden and their impact on health and longevity at high altitudes in a low-income country, potentially guiding future mechanistic and intervention studies on aging in high-altitude environments.

## Result

### Lower risk exposure levels at higher altitude regions of Ethiopia

**Fig. 1:**
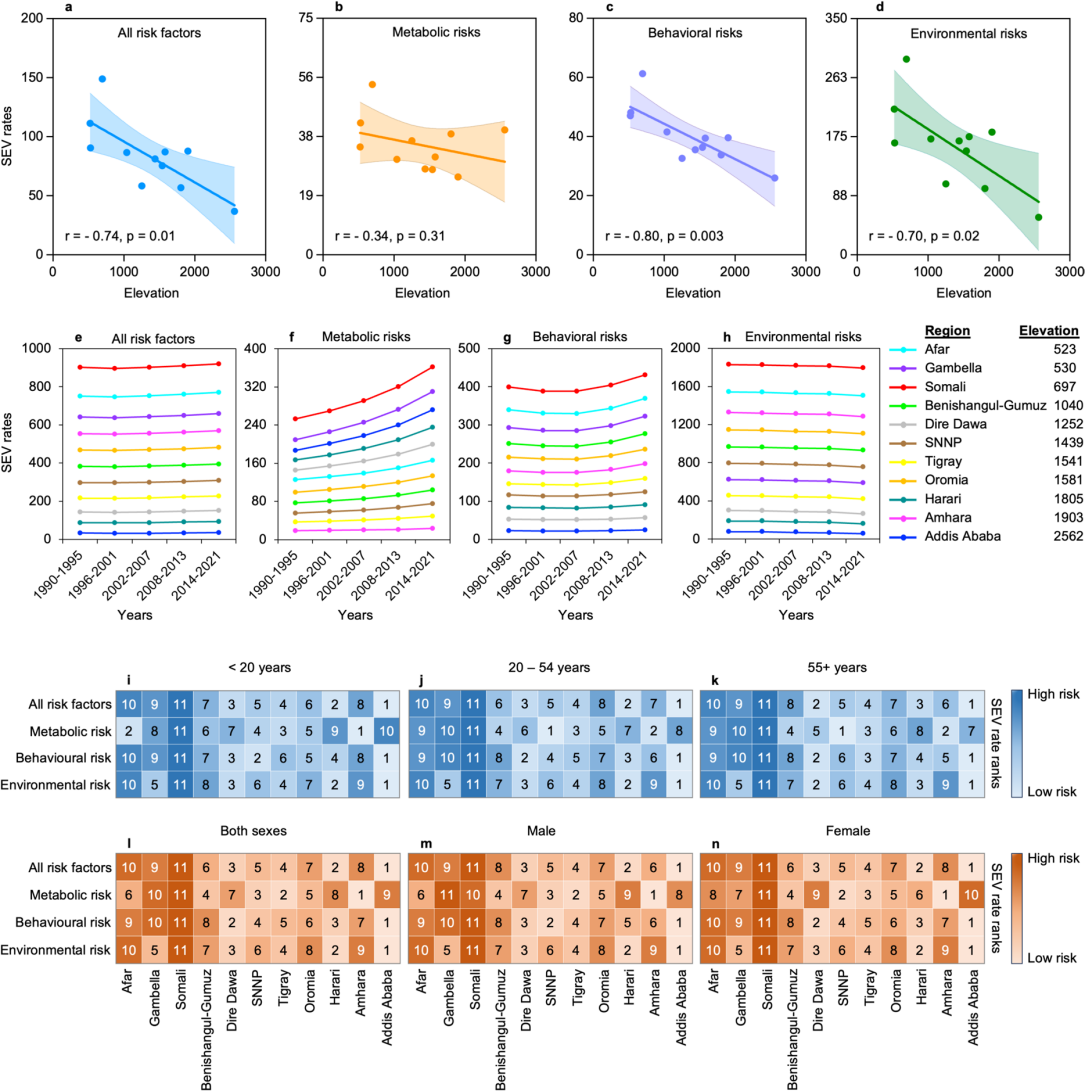
Risk exposure levels in low and high altitude regions of Ethiopia. **a**–**d** Elevation-based variation in risk exposure levels. **e**–**h** Changes in risk exposure levels over 30 years from 1990 to 2021. **i**–**k** Age-specific difference in risk exposure levels. **l**–**n** Magnitude of risk exposure levels in males, females or both

To investigate how regional elevation (Fig. S1) might impact the level of risk factors and population exposure, we mined estimated risk exposure values on all risk factors and on 88 individual risk factors in the form of age-standardized summary exposure value (SEV) rates for the nine regions and two chartered cities of Ethiopia from the GBD Results Tool of the Institute for Health Metrics and Evaluation (IHME). Findings from the GBD 2019 Ethiopia subnational analysis showed the presence of a significant difference in socio-demographic index (SDI) among the Ethiopian regions, and this might impact their risk exposure levels as well. We therefore normalized the SEV values of each region by their corresponding SDI. The normalized SEV rates of each region were then correlated with their corresponding average elevation to assess the impact. As a result, we found a significantly lower rate of age-adjusted SDI normalized summary exposure values for all risk factor (*r* =  − 0.74, *p* = 0.01) as the elevation of the regions increases, indicating overall risk factor level and exposure are lower at higher altitude regions (Fig. 1a). Similar trends were also observed for level 1 risk factors including behavioral (*r* =  − 0.80, *p* = 0.003), environmental (*r* =  − 0.70, *p* = 0.02), and metabolic (*r* =  − 0.34, *p* = 0.31) risk factors (Fig. 1b–d). In contrast, nitrogen dioxide pollution (*r* = 0.62, *p* = 0.04), alcohol use (*r* = 0.12, *p* = 0.73), and ambient particulate matter pollution (*r* = 0.39, *p* = 0.24) have shown increasement at higher altitude regions. Results from correlation of all types of risk factors is found in Supplemental Table 1.

### Lower rates of disease burdenare observed at higheraltitude regions of Ethiopia

**Fig. 2:**
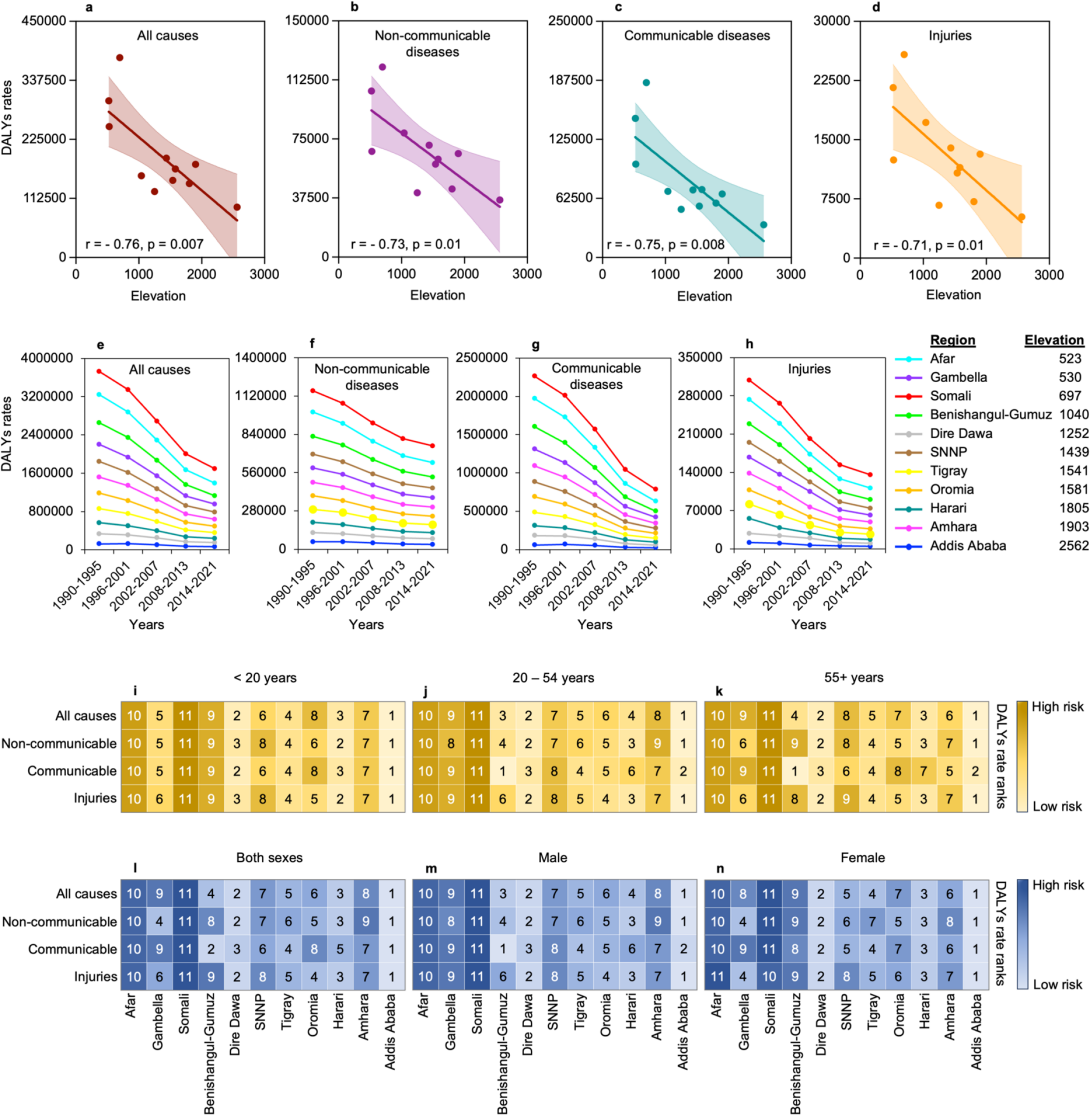
Disease burden in low and high altitude regions of Ethiopia. **a**–**d** Association of disease burden with elevation. **e**–**h** Time-dependent changes in disease burden. **i**–**k** Disease burden in different age groups. **l**–**n** Sex-based variation in disease burden

Regional variation in disease burden and injuries attributed to different altitude levels remained poorly studied. Here we collected rates of age-adjusted disability-adjusted life years (DALYs) as a measure of disease burden from GBD Results Tool of the IHME for the 11 subnational regions of Ethiopia. Estimated DALYs values were obtained for all causes as well as for several distinct causes of disease burden (disease types). We also obtained age-adjusted DALYs rates for each region for the 92 different diseases which the GBD 2017 study on population aging identified as age-related [22]. The effect of regional elevation on disease burden was assessed by correlating the age-adjusted SDI normalized DALYs rates of each region with their average elevation. Our analysis showed a significant decrease in the age-adjusted SDI normalized DALY rates with elevation for all causes combined (*r* =  − 0.76, *p* = 0.007) (Fig. 2a) as well as for all of the level 1 causes including non-communicable diseases (*r* =  − 0.73, *p* = 0.01), communicable disease (*r* =  − 0.75, *p* = 0.008), and injuries (*r* =  − 0.71, *p* = 0.01) (Fig. 2b–d). In contrary, schistosomiasis (*r* = 0.03, *p* = 0.92) and leishmaniasis (*r* = 0.21, *p* = 0.54) have shown an increased trend with elevation although the relationship was not significant (Supplemental Table 2). Similarly, significantly decreased DALYs rates were found for most of the age-related diseases at higher altitude regions of Ethiopia (Supplemental Table 3). In sum, these findings could suggest that high altitude may reduce the pace of aging.

### Longer life expectancy with reduced death rates is observed at high altitude regions of Ethiopia

**Fig. 3:**
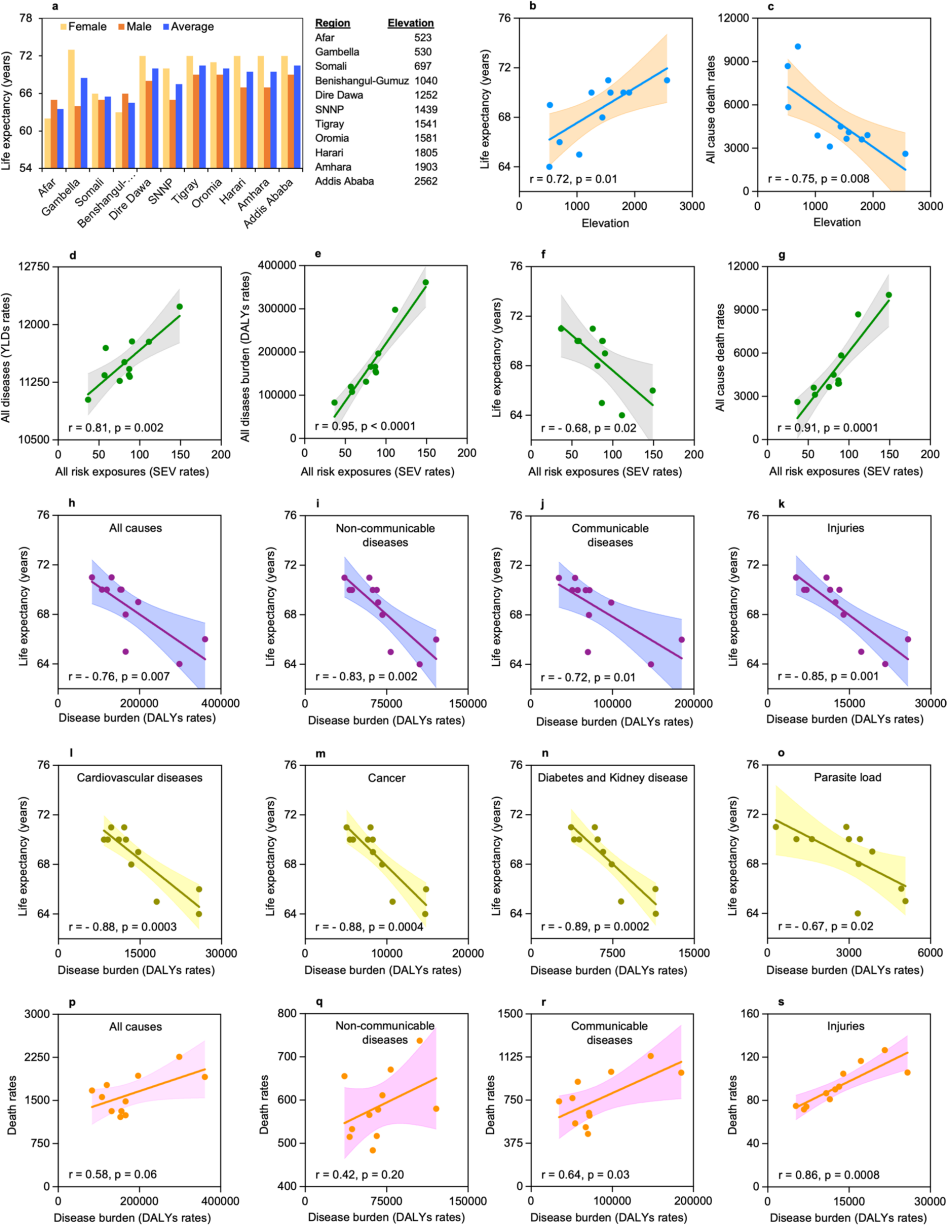
Effect of altitude on disease burden on life expectancy and mortality in high and low altitude regions of Ethiopia. **a** Female, male and average life expectancy in each region of Ethiopia. **b**–**c** Relationship of altitude with life expectancy and death rates. **d**–**g** Effect of risk exposure on disease incidence, disease burden, life expectancy, and mortality rates. **h**–**s** Effect of disease burden due to different causes on life expectancy and death rates

To assess the impact of altitude on human longevity, we correlated the average life expectancy in each region of Ethiopia (Fig. 3a) with their average elevation, and a significantly longer life expectancies were shown at the higher altitude regions (*r* = 0.72, *p* = 0.01) (Fig. 3b). We also investigated differences in mortality rates at higher and lower altitude regions to assess the influence of altitude on lifespan. We found significantly lower death rates at higher altitude regions (*r* =  − 0.75, *p* = 0.008) (Fig. 3c). We further investigated whether the altitude-induced variations in risk exposure impacts disease incidence, disease burden, longevity and lifespan by correlating the SEV rates of all risk factors with years lived with disability (YLDs) rates, all cause DALYs rates, life expectancy, and then all causes death rates of each region. As risk exposure increases, disease occurrence increases (*r* = 0.81, *p* = 0.002) (Fig. 3d) so as disease burden (*r* = 0.95, *p* < 0.0001) (Fig. 3e), leading to shorter lifespan (*r* =  − 0.68, *p* = 0.02) (Fig. 3f) and increased mortality rates (*r* = 0.91, *p* = 0.0001) (Fig. 3g). Moreover, we examined the impact of disease burden on life expectancy and mortality. The average life expectancy significantly decreases as the disease burden due to all causes (*r* =  − 0.76, *p* = 0.007) (Fig. 3h), non-communicable diseases (*r* =  − 0.83, *p* = 0.002) (Fig. 3i), communicable diseases (*r* =  − 0.72, *p* = 0.01) (Fig. 3j), and injuries (*r* =  − 0.85, *p* = 0.001) (Fig. 3k) increases. A similar significant decrease in life expectancy was observed specifically due to cardiovascular diseases (*r* =  − 0.88, *p* = 0.0003) (Fig. 3l), cancer (*r* =  − 0.88, *p* = 0.0004) (Fig. 3m), and diabetes and kidney disease (*r* =  − 0.89, *p* = 0.0002) (Fig. 3n) as well as parasitic infections (*r* =  − 0.82, *p* = 0.002) (Fig. 3o), which are the leading non-communicable and communicable diseases in Ethiopia. In contrast, the mortality rates showed an increasing trend as the disease burden attributed to all causes (*r* = 0.58, *p* = 0.06) (Fig. 3p), non-communicable diseases (*r* = 0.42, *p* = 0.20) (Fig. 3q), communicable diseases (*r* = 0.64, *p* = 0.03) (Fig. 3r), and injuries (*r* = 0.86, *p* = 0.0008) (Fig. 3s) increases, and only significant for communicable diseases and injuries.

### Increased photoaging is observed at higher living altitudes of Tigray in Ethiopia

**Fig. 4:**
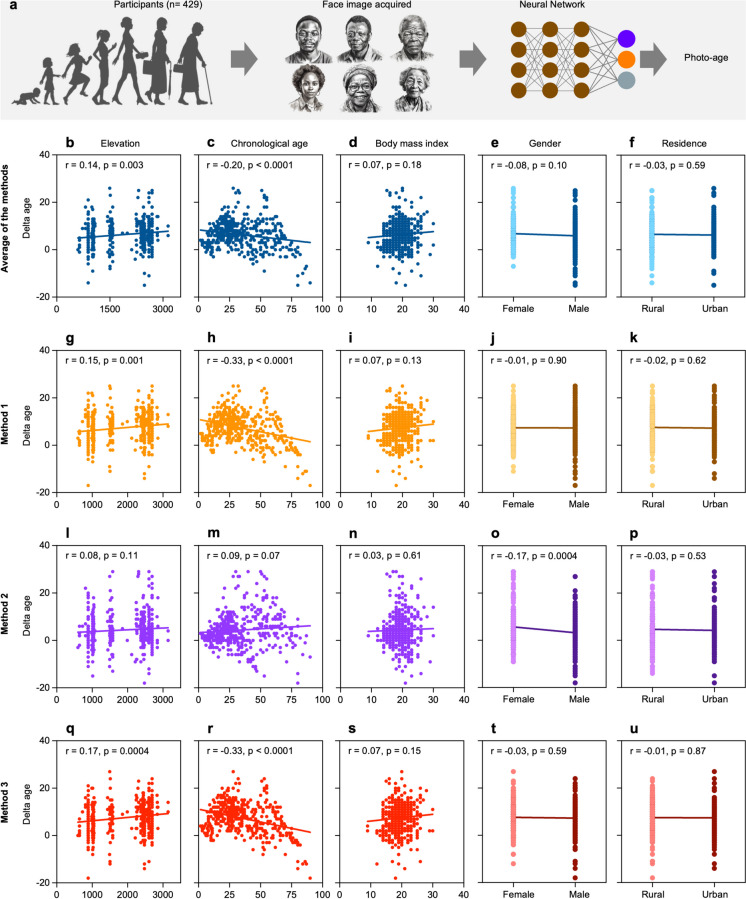
Rate of biological aging at different elevation levels in Tigray. **a** Workflow showing age prediction from facial images of the lowland and highland participants (n.b. photos are AI generated and do not reflect real individuals). **b**–**u** Scatter plots of the different models versus indicated parameters of the study participants

To gain insight into whether a varied biological aging is observed in response to exposure to high altitude chronic hypoxia and other related factors, we investigated aging biomarkers of the highland and lowland dwellers in the Tigray region in Northern Ethiopia (Fig. S3). Given the challenging conditions of working in the less developed areas, biomarkers were chosen that could be collected with relative ease. Previous studies have suggested that facial images can be used as a biomarker to estimate biological aging [23]. Based on this, we cross-sectionally collected facial images from a cohort of 429 participants dwelling at varying altitudes (600 to 3200 masl) in the Tigray region of Northern Ethiopia (Fig. 4a, Table 1). We applied four different deep learning algorithms (Fig. S3) to these images and generated predicted age for each participant using the three algorithms that had the most accurate predictive age association (Fig. S4). The age difference calculated by subtracting the chronological age from the predicted age was correlated with the average residential elevation of each participant, and we found that the rate of facial aging generally increases as the living altitude of the participants increases (*r* = 0.14, *p* = 0.0027) (Fig. 4b). However, a decreased aging rate was shown as the chronological age of the participants increases (*r* =  − 0.20, *p* < 0.0001) (Fig. 4c). We also investigated the impact of body mass index, sex, and residence on the rate of aging of the participants, and no significant effect of these factors was found on the rate of aging of the participants (Fig. 4d–f).

**Table 1:** Covariate information of the study participants

Characteristics of the study sites	Lowland				Highland					
	Sheraro	Dedebit	Alamata	Combined	Hagereselam	Edagahamus	Korem	Maychew	Combined	*P value*
	(*n* = 105)	(*n* = 50)	(*n* = 47)	(*n* = 202)	(*n* = 51)	(*n* = 50)	(*n* = 54)	(*n* = 72)	(*n* = 227)	
Average elevation (masl)	988	912	1544	1093	2534	2689	2446	2481	2537	< 0.001
Chronological age	31 ± 18	37 ± 24	37 ± 17	34 ± 20	31 ± 14	36 ± 18	27 ± 16	40 ± 24	33 ± 19	0.6
Sex										
Female	50	21	25	96	24	23	28	31	106	
Male	55	29	22	106	27	27	26	41	121	
Residence										
Urban	75	0	37	112	31	33	33	50	147	
Rural	30	50	10	90	20	17	21	22	80	
Height (m)	155 ± 23	158 ± 15	165 ± 8	158 ± 19	161 ± 17	158 ± 16	152 ± 16	159 ± 14	157 ± 16	0.49
Weight (kg)	49 ± 15	47 ± 14	54 ± 10	50 ± 14	48 ± 11	49 ± 13	45 ± 13	50 ± 14	48 ± 13	0.23
Body mass index (kg/m^2^)	19 ± 3	19 ± 3	20 ± 3	19 ± 3	19 ± 3	19 ± 3	19 ± 3	20 ± 4	19 ± 3	0.57
Resting metabolic rate (calories/day)	1372 ± 233	1350 ± 199	1440 ± 170	1383 ± 213	1404 ± 222	1373 ± 208	1325 ± 202	1376 ± 205	1370 ± 210	0.53

### Reduced predicted DNA damage-induced senescence was observed at higher altitudes of Tigray in Ethiopia

**Fig. 5:**
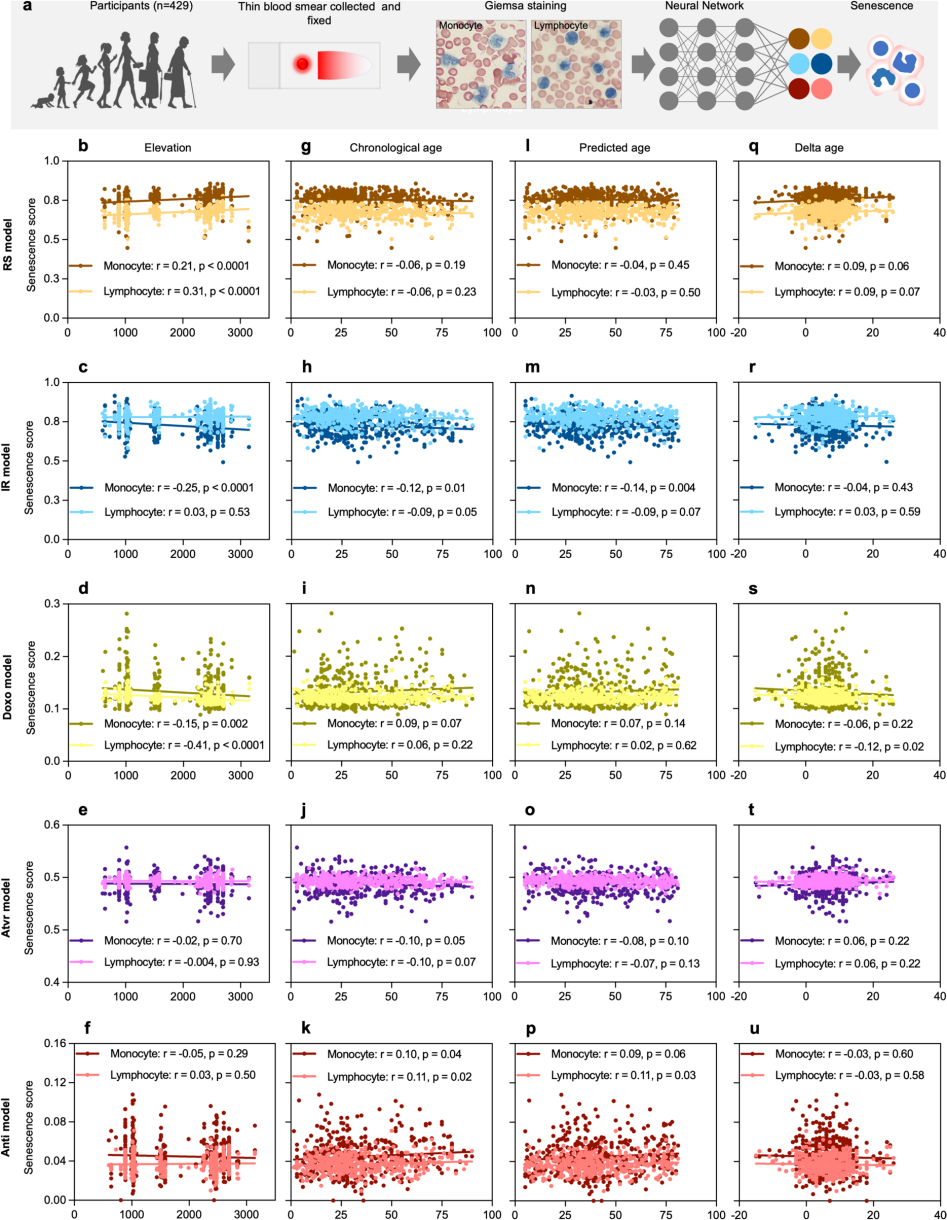
Senescence status of highland and lowland dwellers. **a** Workflow of senescence prediction from nuclear morphology of PBMCs. **b**–**u** Scatter plots of the different models versus indicated parameters of the study participants

Nuclear morphology has been shown to be a biomarker of senescence [24]. Thus, in addition to the facial images, we also collected images of peripheral blood mononuclear cells (monocytes and lymphocytes) using a Giemsa stained thin-layer blood smears from the cohort of 429 participants. To investigate senescence, we identified nucleated cells in the blood smears of the highland and lowland dwellers and then applied the nuclear senescence predictor (NUSP) (Fig. 5a). We used five different NUSP models to generate senescence scores and correlated these with the participants' living altitude to assess the effect of high altitude on nuclear senescence of the immune cells (Fig. 5b–u). Notably, our analysis showed significantly higher senescence scores with elevation using the replicative exhaustion (RS) for both monocytes (*r* = 0.21, *p* < 0.0001) and lymphocytes (*r* = 0.31, *p* < 0.0001) (Fig. 5b), but significantly lower senescence scores for monocyte (*r* =  − 0.25, *p* < 0.0001) using the ionizing radiation (IR) model (Fig. 5c) as well as monocytes (*r* =  − 0.15, *p* = 0.002) and lymphocytes (*r* =  − 0.41, *p* < 0.0001) using the doxorubicin (Doxo) model (Fig. 5d). In addition, increasing chronological age was significantly negatively correlated with the senescence scores detected by the IR model (monocytes: *r* =  − 0.12, *p* = 0.01; lymphocytes: *r* =  − 0.09, *p* = 0.049) (Fig. 5h), but significantly positively correlated with the senescence scores from the Anti model (monocytes: *r* = 0.10, *p* = 0.04; lymphocytes: *r* = 0.11, *p* = 0.02) (Fig. 5k). Examining the effect of increasing predicted age, we found lower scores for the senescence of monocytes detected by the IR model (*r* =  − 0.14, *p* = 0.004) (Fig. 5m), whereas higher scores for the senescence of lymphocytes were detected by the Anti model (*r* = 0.11, *p* = 0.03) (Fig. 5p). Rate of aging of the participants seems less important to affect monocyte senescence, but for lymphocytes, the Doxo model showed lower scores as the rate of aging of the participants increases (*r* =  − 0.12, *p* = 0.02) (Fig. 4s). Furthermore, we have investigated how the senescence status of the PBMCs is affected by the body mass index, sex, and rural or urban residence of the participants (Fig. S5). As a result, detection by the Atvr (mitochondrial dysfunction) model showed lower senescence scores of monocytes in the participants with higher body mass index (*r* =  − 0.12, *p* = 0.03) (Fig. S5j).

### Increased effect of resting metabolic rates (RMR) on photoaging and replicative associated senescence of lymphocytes

**Fig. 6:**
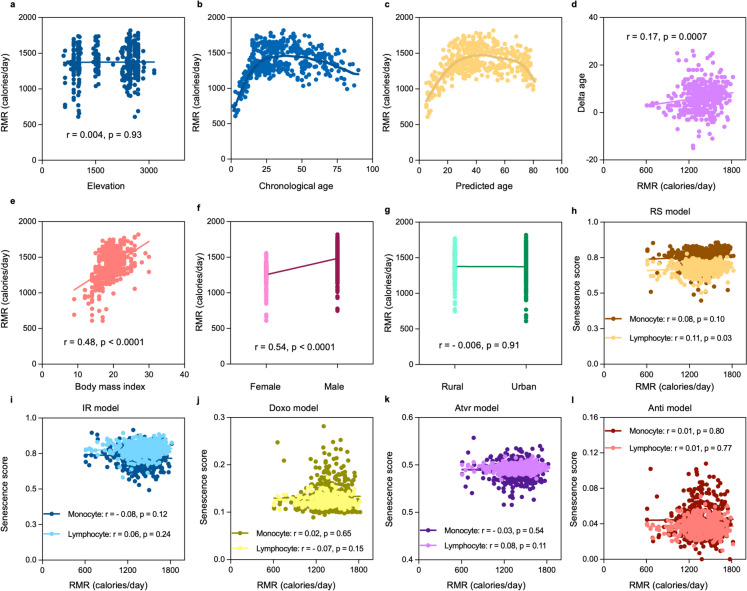
Resting metabolic rate of highland and lowland dwellers and its effect on the rate of aging and senescence. **a**–**g** Relationship of resting metabolic rate (RMR) with elevation, chronological age, predicted age, delta age, body mass index, gender, and residency respectively. **h**–**l** Relationship of RMR with senescence of monocytes and lymphocytes

Differences in aging rate could be related to the metabolic activity of the body [25]. To gain clues whether there is a difference in energy expenditure between the highland and lowland dwellers and if this impacts their aging rate, we calculated the resting metabolic rate (RMR) using the Revised Harris-Benedict equation [26], and correlated this to their dwelling altitude. As a result, no variation in energy consumption was shown linked to the participants's dwelling altitude (*r* = 0.004, *p* = 0.93) (Fig. 6a). We then correlated the RMR with chronological and predicted age to assess its effect on the rate of aging. As expected, RMR does not appear to fit to a linear curve and appears to increase until age 20 where it plateaus and at old age declines slightly (Fig. 6b, c). Surprisingly, RMR correlates positively with delta age perhaps indicating that increased metabolic rate is associated with increased speed of aging (*r* = 0.17, *p* = 0.0007) (Fig. 6d). However, as described above, delta age was also correlated with BMI which may contribute to this relationship. We further assessed the impact of body mass index, sex, and residency on the RMR of the participants, and an increased RMR was observed in participants with higher body mass index (*r* = 0.48, *p* < 0.0001) (Fig. 6e) and male participants (*r* = 0.54, *p* < 0.0001) (Fig. 6f). Additionally, we examined the effect of RMR on the different senescence types, and we found a significant effect on RS of lymphocytes (*r* = 0.11, *p* = 0.03) (Fig. 6h–l).

## Conclusion

Alterations in oxygen levels have been associated with health and disease [27]. Our analysis revealed that increased altitude affects a wide range of risk factors and diseases as well as rate of biological aging, which in turn affects life expectancy and mortality rates. Notably, we saw a strong decrease in disability adjusted life-years with increasing life expectancy and lower death rates suggesting that increased altitude is associated with better health. This supports previous findings that people at highlands live longer and often have better health than those living at lowlands [28]. Other studies have, however, found an association of high altitude with higher prevalence of cancer and cancer attributable death rates [29, 30], in particular skin cancer [31, 32]. This is in agreement with our deep learning investigations on facial images that showed a faster rate of aging of highland dwellers than the lowland dwellers, and this is likely attributed to the high level of ultraviolet radiation at high altitude [33, 34]. However, the resting metabolic rate has shown a strong positive association with the rate of aging although it remains unknown how this drives biological aging. Given the role of oxygen on driving oxidative stress, we speculated that increased altitude might affect senescence. Accordingly, we found a reduction in DNA damage-related senescence (IR and Doxo), suggesting a protective role of high altitude in some aspects of biological aging. Surprisingly, we saw increased replicative associated senescence with increasing altitude suggesting that there may be phenotypical overlap between previously described hypoxia-induced senescence and replicative senescence. This could also be associated with how the body uses its energy as the replicative associated senescence specifically of lymphocytes showed positive relationship with resting metabolic rates. Accordingly, chronic hypoxia in sleep apnea patients leads to shortened telomeres [35]. Taken together, our analyses showed that high altitude may be a key factor in modulating not only aging-associated risk factors and diseases but also biological aging. Overall, our study represents a novel effort in understanding the biological aging of the unique population of high-altitude residents of a low income country. Nurturing the efforts to understand the mechanism of survival and aging process at high altitude would generate knowledge that may benefit the global population in the prevention and treatment of disabilities and illnesses.

Our study has several limitations. For instance, to study the levels of risk factors and disease burden at different altitudes in Ethiopia, we used data from the Institute for Health Metrics and Evaluation. In Ethiopia, although currently improving, the data reporting system in some areas is still not technologically supported, and in such circumstances, the reporting is done manually, making it prone to mis- or overreporting or data loss leading to data bias. Further, in our study, we only involved volunteers who are healthy and permanent or long-term residents in their territory. The healthy status of each participant was based on their declaration and based on the physical examination performed by the licensed health professional, and this may not reflect the biological situation of the participants. The residence history of the participants was also based on what they reported, and to minimize effects of changing residence from low- to highland or vice versa. Our biomarker investigations have been done using largely noninvasive methodologies. Consequently, the limitations of these systems may have affected our results as well. We tried to minimize such effects by combining different methods to produce averaged data for analysis and presentation. In fact, a strong association is observed among predicted age by most of the different age predictor tools we used, suggesting low variation by methods. Further, senescence scores of each highland and lowland participant were deduced from stained nuclear morphology of the immune cells. Despite color normalization, differences in staining intensities could subtly affect the generated senescence scores. To avoid such variation, we performed the staining of all smears of all participants simultaneously. In addition, during analysis, intensity normalization was performed at the individual level and between the lowland and highlanders. It is also important to underline that the microscopic identification of lymphocytes and monocytes from other blood cells in the smears was performed manually and based on cytoplasmic and nuclear characteristics, indicating the possibility of involving other cells that morphologically resemble monocytes or lymphocytes. To minimize this, the detection and capturing of monocytes and lymphocytes from the thin blood smear was performed by a licensed Medical Laboratory Technologist. It is also possible that the deep learning algorithms may have reduced accuracy in assessing senescence in blood smears. Nevertheless, it is relatively striking that two different predictors describe decreased DNA damage-induced senescence with elevation.

Overall, our study has provided a comprehensive analysis about the relationship of altitude with risk exposure and diseases burden and the influence of these relationships on the healthspan and lifespan of Ethiopians. Our study also investigated the biomarkers of the lowland and highland dwellers to characterize biological aging at high and low dwelling altitudes. Taken together, these analyses indicated that high altitude is associated with lower risk exposure, lower disease burden and lower DNA damage-induced senescence. Our study also demonstrated the value of deep learning tools in research in areas where research facilities are limited such as in Ethiopia. And our works has provided pioneering insight about the population aging at high altitude.

## Methods

### *Study area and setting*

Ethiopia is an East African country with an economic and social development classified among the least developed countries (LDCs) [36]. Being the second most populous nation in Africa (estimated 109.5 million in 2024) [37, 38], Ethiopia represents the highest proportion of native highland dwellers in the world [12]. In chronological perspective, the Ethiopian population is young (median age 18.9) and the elderly population (65 years and above) accounted for 5.1% in 2010 and estimated to reach 10.3% by 2050 [39]. The Federal Democratic Republic of Ethiopia has been administratively divided into 11 subnational regions, namely, Tigray, Amhara, Afar, Somali, Harar, Dire Dawa, Oromia, Addis Ababa, Gambella, Benshangul Gumuz, and South Nations Nationalities and People (SNNP). Interestingly, these subnational regions have highly varied mean elevation (Fig. S1a–b), making it very suitable for investigating the impact of regional variation in elevation on risk exposure levels and disease burden as well as human aging. Further, within specific region such as Tigray, there exists a large difference in living altitude (600 to 3200 masl) (Fig. S1c–d). Consequently, we chose the Tigray region to examine the impact of high altitude on human aging. Besides, the Tigray region has millions of people permanently dwelling at both higher and lower altitudes (Fig. S2a–g), and these lowland and highland populations have similar sociodemographic index [40]. To gain the most possible impact of high altitude on human aging, we selected participants from residential areas with the highest mean elevation in Tigray including Hagereselam, Edagahamus, Korem, and Maychew. Sheraro, Dedebit, and Alamata are among the lowland residential areas in Tigray.

### Data source for risk exposure and disease burden assessments

To investigate elevation-based regional variation in risk exposure levels, disease burden, life expectancy, and mortality rates in Ethiopia, estimated rates of age-adjusted summary exposure value (SEV) as a measure of risk exposure, estimated rates of age-adjusted years lived with disability (YLDs) as a measure of healthy life lost due to diseases, estimated rate of age-adjusted disability-adjusted life years (DALYs) as a measure of disease burden, and age-adjusted death rates were minded from the Global Health Data Exchange (GHDx) of the Institute for Health Metrics and Evaluation [41] for each of the 11 subnational regions (nine regions and two chartered cities) of Ethiopia for the years 1990 to 2021. SEV data was collected for all risk factors as well as for level 1, level 2, level 3, and level 4 risk factors for each region. Similarly, YLDs, DALYs, and death rates were obtained for all causes and level 1, level 2, level 3, and level 4 causes for each region. In addition, to assess the impact of risk exposure and disease burden on longevity, life expectancy data was collected from the National Data Management Center for Ethiopia for the year 2019 [42]. The mean elevation of each region was determined and provided by the Tigray Statistical Agency (Tigray, Ethiopia). Data about the socio-demographic index (SDI) of each region was gathered from the GBD 2019 Ethiopia subnational analysis [43].

### Human participants for biological aging studies

We conducted a cross-sectional clinical trial to examine the biological aging of highland and lowland populations in the Tigray region of Northern Ethiopia (ethical approval number: MU-IRB 2020/2022, see below). Four hundred twenty-nine volunteer participants were recruited, of which 53% (229) were native highland dwellers and 202 were lowland dwellers. All participation was voluntary and after informed consent provided. From all participants, we collected covariate data, facial images, and thin blood smears from April to May 2022.

### Sociodemographic data collection

We collected demographic data regarding the chronological age, sex, residence, ethnicity, movement history, and health status of the study participants using a self-developed short questionnaire. The height and weight of each participant were recorded from stadiometer and digital scale, respectively. Body mass index (BMI) was calculated using the measured height and weight of each participant as previously described [44]. Similarly, the Revised Harris-Benedict formula was utilized to calculate the resting metabolic rate (RMR) of each participant [26]. Elevation data of the residential area (locally known as “Tabiya”) of each participant was obtained from the Tigray Statistical Agency.

### Inclusion criteria

We involved residents who voluntarily provided their informed consent to participate in this study. In addition, it was a requirement that the volunteer was a permanent resident in a given place or moved to another place within Tigray, but with the same elevation. This was to ensure that the highland participants were not exposed to normoxia, or the lowland participants were not exposed to high-altitude hypoxia. Further, we considered only those volunteers who had self-declared that they had been healthy for the previous 6 months and had no finding on physical examination by the healthcare professional.

### Face image collection

We acquired facial images from all the study participants using the Samsung Galaxy A9 mobile phone (Samsung, Denmark). The mobile is fitted with a 24 mp camera with capability to self-adjust brightness, color, and contrast to produce high dynamic range (HDR) images. Up to ten-colored, 3 × 4 sized HDR images were captured at white background from each participant. After acquisition, the images were checked for their quality, and bad quality images such as with unintended brightness, contrast, or coloration or images that do not meet the requirements for image analysis by artificial intelligence tools were discarded. Correctly acquired images were annotated with location and participant information to ensure where and from whom they were collected. Subsequently, the images were transferred to and stored on password protected personal computers and electronic storage devices. After we completed image collection from all the study participants, we performed de-identification by removing all the identifiers of the participants and the images were coded to ensure confidentiality.

### Facial image-based age prediction

We used four different freely online available AI methods to predict age of each study participant from their facial images. These are method 1 (https://saas.haut.ai/), method 2 (https://www.facialage.com/), method 3 (https://howolddoyoulook.com/), and method 4 (https://age.toolpie.com/). Each method is built-in with algorithms to perform a step-by-step analysis regarding the uploaded image quality and features in the face skin to produce estimated age. Using each predictor, we performed four independent analyses on four different face images of each participant and produced four independent predicted ages per participant. We performed principal component analysis on the predicted ages to examine the variation among these methods (S3). Method 1 was excluded and the predicted ages of each participant from the three methods (methods 2 to 4) and the average of them were used in further analysis.

### Thin blood smear collection and staining

After we captured face images, we subsequently collected peripheral blood smears from each highland and lowland participant. Up to five thin blood smears per participant were prepared from the middle fingertip by finger prick. Air-dried smears were fixed in absolute methanol and stained in freshly prepared Giemsa stain as previously described [45]. To avoid in-group and between-group staining variation, both highland and lowland smears were stained in the same working Giemsa solution, simultaneously.

### Peripheral blood mononuclear cell (PBMC) image acquisition

We examined the Giemsa-stained blood films under oil immersion objective of the OPTIKA light microscope (Optika, Italy) for the detection of peripheral blood mononuclear cells (PBMCs). The microscope is fitted with a camera and linked to Optika Vision Pro version 2.7 software on a desktop where the images of PBMCs are displayed and captured. Peripheral blood mononuclear cells include monocytes and lymphocytes, and detection and identification of these cells from each other or from other blood cells was done based on cell size, nuclear shape, granulation, and/or staining properties [45]. We captured over 100 images of PBMCs per participant (70% lymphocytes), and more than 43,000 images from all the study participants.

### Peripheral blood mononuclear cell (PBMC) senescence prediction

We used five different deep learning models to detect senescence in monocytes and lymphocytes of each participant. Each of these models is specialized to recognize a specific type of senescence. Notably, the RS model is specific to detect senescence due to replicative exhaustion, whereas the IR and Doxo models are trained on senescent cells from DNA damage attributed to ionizing radiation (IR) and doxorubicin (Doxo) treatment, respectively. The Anti and Atvr models detect senescence caused by antimycin-A-induced mitochondrial dysfunction and atazanavir/ritonavir-induced proteotoxicity, respectively [46]. More than 43,000 captured images of the PBMCs (~ 70% lymphocytes) were uploaded to each model, and the model-specific algorithms analyze the nuclear morphology of the PBMCs and generated cell type-specific senescence scores for each participant [24].

### Statistical analysis

IBM SPSS Statistics Version 29.0.1.0 (171) for Mac OS was used to determine the correlation of risk factors and disease burden with elevation. Graphs and heatmaps showing the trend and rank of risk factors and disease burden were done in Microsoft Excel 365. All scatter plots and linked data processing, statistical analysis, and graphs were made utilizing GraphPad Prism Version 10.1.1 for Mac OS (GraphPad Software, Boston, MA, USA). The descriptive statistics of the continuous variables of the study participants were presented as mean ± standard deviation, all other variables, as counts. Delta age or age difference or aging acceleration or deceleration was calculated by subtracting the chronological age from the predicted age. Simple linear regression and Pearson’s correlation as well as lowess curve were applied to determine all the relationship studies. In all analyses, significance was considered at *p*-values less than 0.05.

## Supplementary Information

Below is the link to the electronic supplementary material.

Supplementary file1 (DOCX 61216 KB)

Supplementary file2 (XLSX 28 KB)

## Data Availability

Data not in this article are included as supplementary material. If further data is required, inquiries can be forwarded to the corresponding author.
