# Supplementary material for: Deep learning reveals diverging effects of altitude on aging
Source: GeroScience. 2025 Jan 15;47(3):3873–89. doi: 10.1007/s11357-024-01502-8 (PMC12181603; doi:10.1007/s11357-024-01502-8)
Supplement: Supplementary file 1 — Supplementary file1 (DOCX 61216 KB) [file 11357_2024_1502_MOESM1_ESM.docx]

Supplemental figures for

**Deep learning reveals diverging effects of altitude on biological aging**

Amanuel Abraha Teklu^1,2^, Indra Heckenbach^1^, Michael Angelo Petr^3^, Daniela Bakula^1^, Guido Keijzers^1^, Morten Scheibye-Knudsen^1*^

^1^Center for Healthy Aging, Department of Cellular and Molecular Medicine, University of Copenhagen, Copenhagen, Denmark

^2^Department of Biochemistry and Molecular Biology, College of Health Sciences, Mekelle University, Ethiopia

^3^Tracked Biotechnologies, LLC, Manassas, Virginia, USA

*Correspondence: mscheibye@sund.ku.dk

**Contains:**

Supplemental figures 1-5

**Figure S1: Study area and setting to investigate risk exposure, disease burden and human aging at varying elevation levels in Ethiopia.** **a** Elevation map of Ethiopia and its subnational regions. **b** Mean elevation and elevation-based changes in the partial pressure of oxygen in the inspired air of the subnational regions of Ethiopia. **c** Facial images and blood smears collection sites in Tigray. **d** Mean elevation of the sample collection sites with estimated inhaled oxygen concentration. The elevation map of Ethiopia and of the Tigray region was made and provided by the Tigray Statistical Agency in Tigray, Ethiopia. The barometric pressure and the partial pressure of oxygen in the inspired air at subnational and study sites levels were calculated as previously described (11): Barometric pressure = 760 × [1 − 0.0065 × elevation in meters/(273.15 + temperature)]5.255 , PiO2 = (barometric pressure – 47 mmHg) × 0.2093


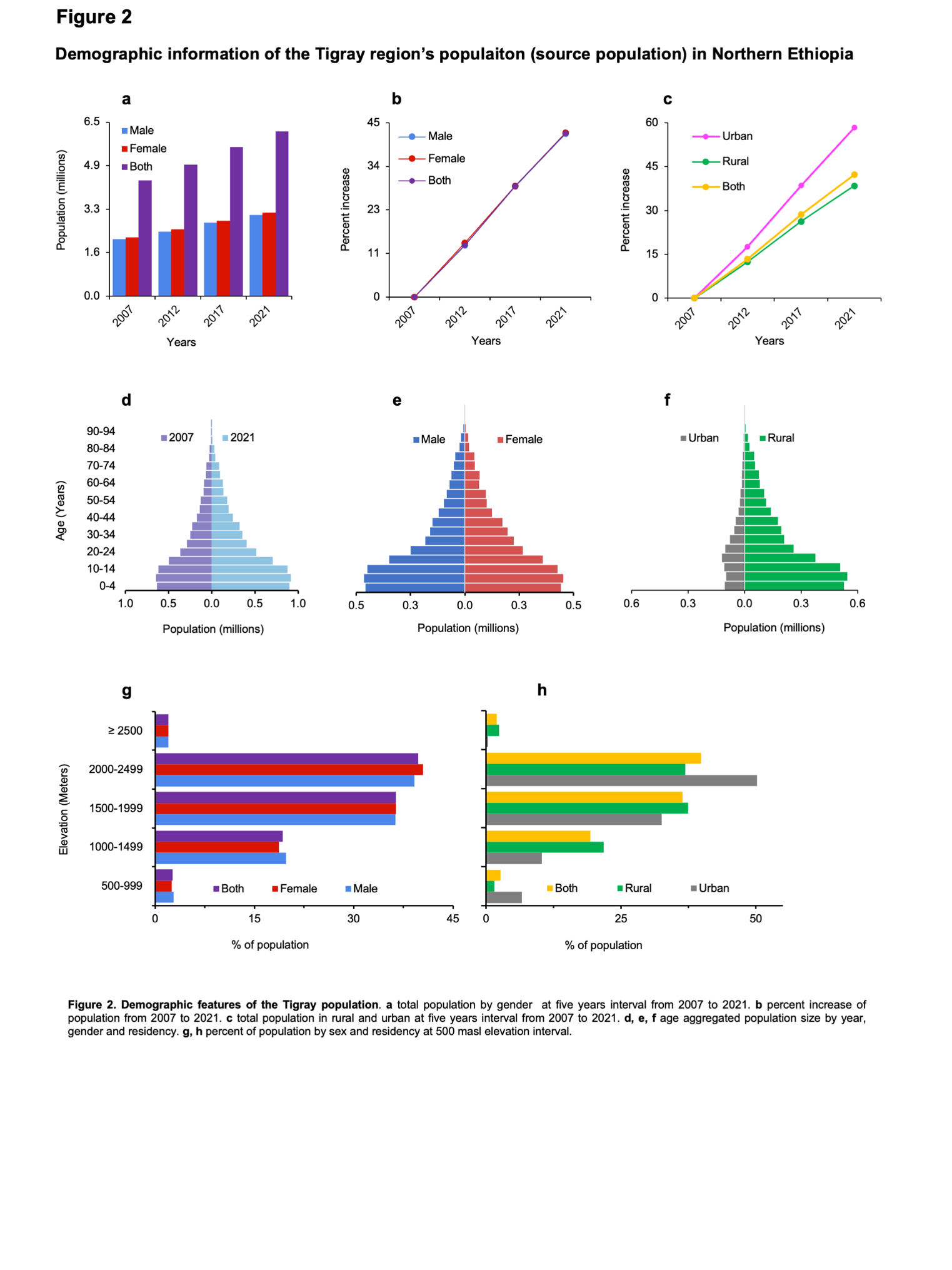


**Figure S2: Demographic features of the Tigray population. a** Total population by gender at five years interval from 2007 to 2021. **b** Percent increase of population from 2007 to 2021. **c** Total population in rural and urban at five years interval from 2007 to 2021. **d** Age aggregated population size by year. **e** Age aggregated population size by gender. **f** Age aggregated population size by residency. **g** Percent of population by sex at 500 masl elevation interval. **h** Percent of population by residency at 500 masl elevation interval.


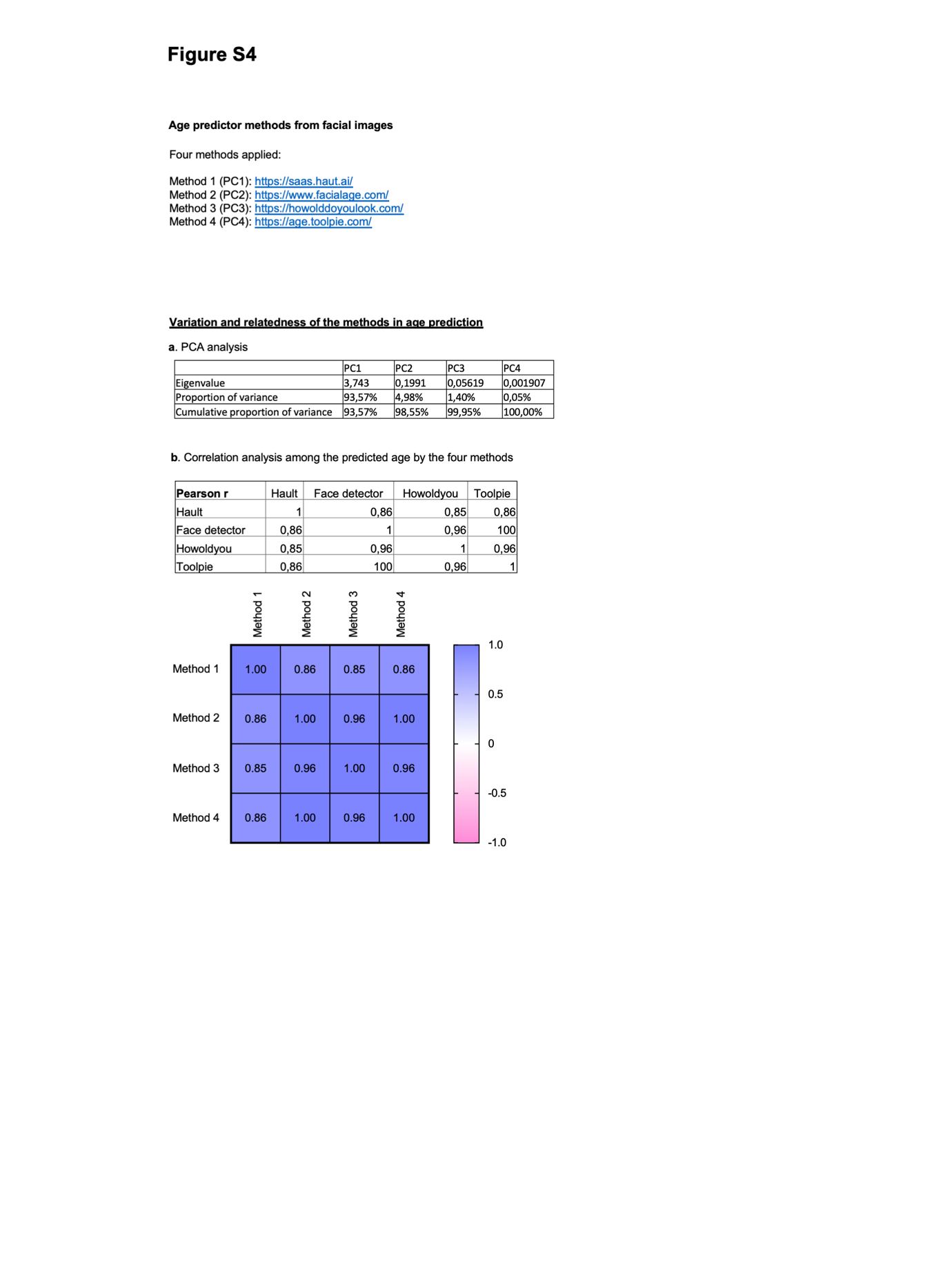


**Figure S3: Variation and relatedness of age predictor methods from facial images**. **a** PCA or predicted age by the four different age predictors. **b** Correlation result of the predicted age by the four different age predictors.


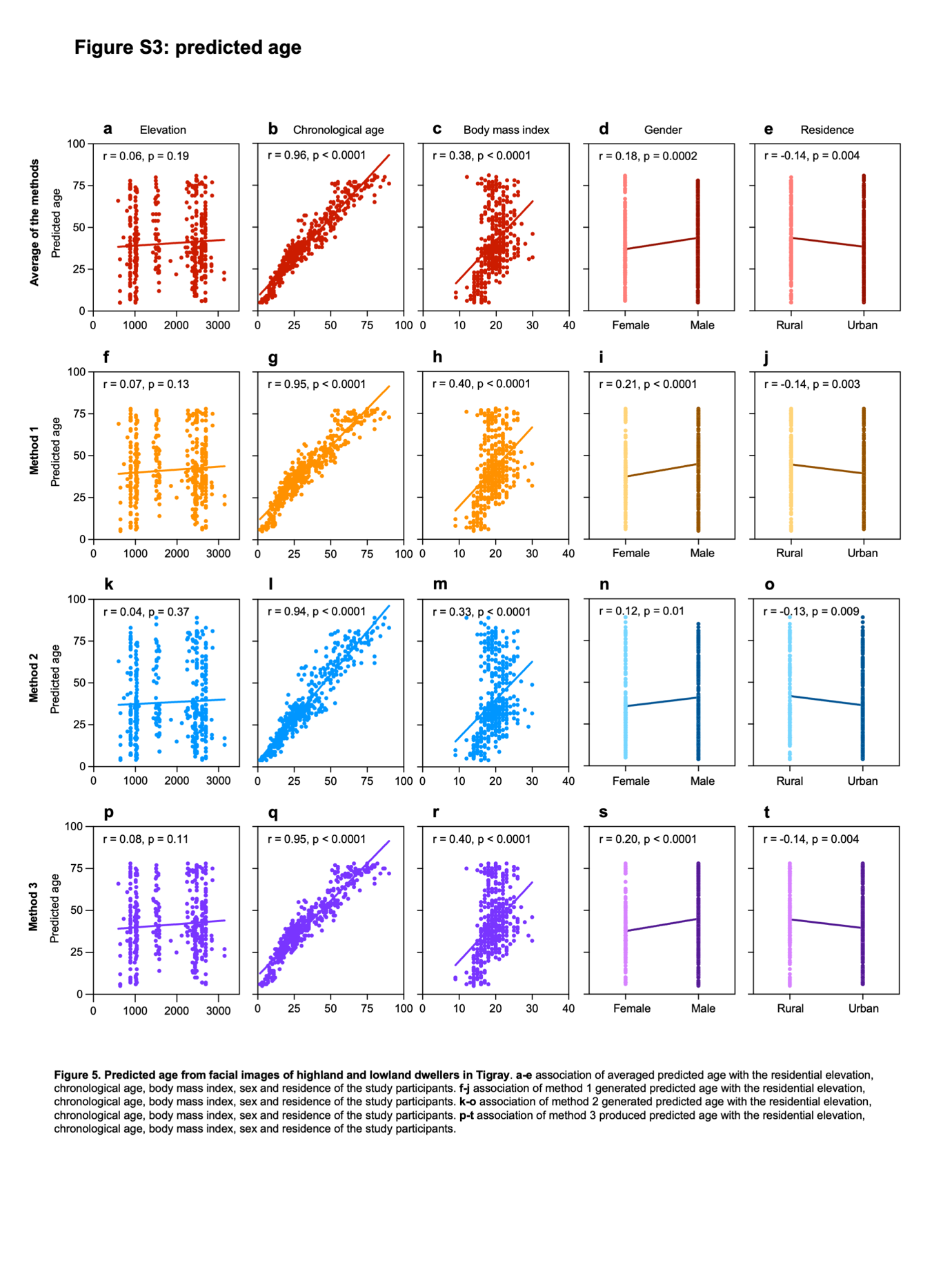


**Figure S4: Predicted age from facial images of highland and lowland dwellers in Tigray. a** Scatter plot of averaged predicted age with the residential elevation of the participants. **b** Scatter plot of averaged predicted age with the chronological age of the participants. **c** Scatter plot of averaged predicted age with the body mass index of the participants. **d** Scatter plot of averaged predicted age with the gender of the participants. **e** Scatter plot of averaged predicted age with the residence of the participants. **f** Scatter plot of predicted age by method 1 with the residential elevation of the participants. **g** Scatter plot of predicted age by method 1 with the chronological age of the participants. **h** Scatter plot of predicted age by method 1 with the body mass index of the participants. **i** Scatter plot of predicted age by method 1 with the gender of the participants. **j** Scatter plot of predicted age by method 1 with the residence of the participants. **k** Scatter plot of predicted age by method 2 with the residential elevation of the participants. **l** Scatter plot of predicted age by method 2 with the chronological age of the participants. **m** Scatter plot of predicted age by method 2 with the body mass index of the participants. **n** Scatter plot of predicted age by method 2 with the gender of the participants. **o** Scatter plot of predicted age by method 2 with the residence of the participants. **p** Scatter plot of predicted age by method 3 with the residential elevation of the participants. **q** Scatter plot of predicted age by method 3 with the chronological age of the participants. **r** Scatter plot of predicted age by method 3 with the body mass index of the participants. **s** Scatter plot of predicted age by method 3 with the gender of the participants. **t** Scatter plot of predicted age by method 3 with the residence of the participants.


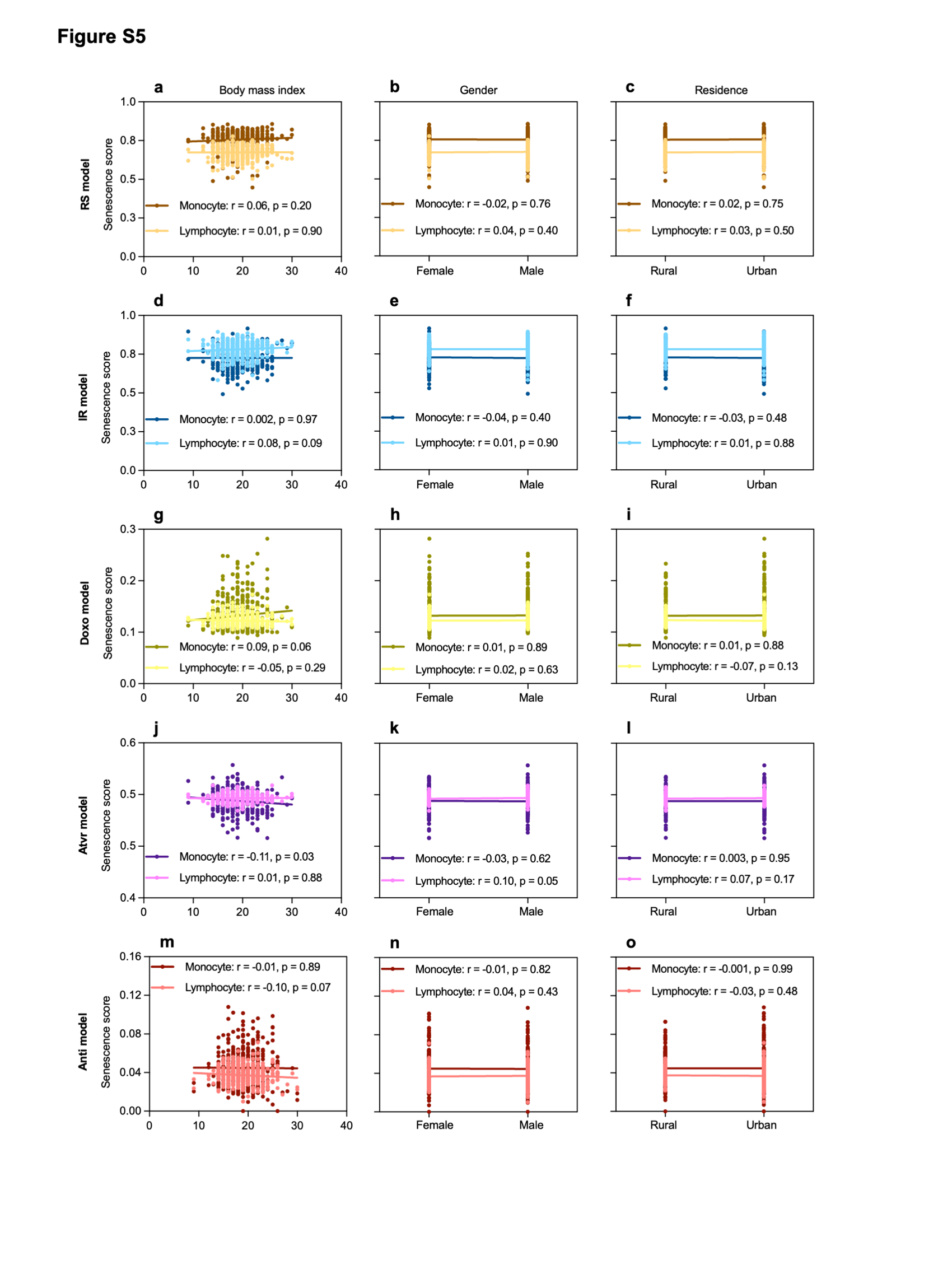


**Figure S5: Senescence status of highland and lowland dwellers**. **a** Scatter plot of predicted senescence (RS model) of monocytes and lymphocytes with the body mass index of the study participants. **b** Scatter plot of predicted senescence (RS model) of monocytes and lymphocytes with the gender of the study participants. **c** Scatter plot of predicted senescence (RS model) of monocytes and lymphocytes with the residence of the study participants. **d** Scatter plot of predicted senescence (IR model) of monocytes and lymphocytes with the body mass index of the study participants. **e** Scatter plot of predicted senescence (IR model) of monocytes and lymphocytes with the gender of the study participants. **f** Scatter plot of predicted senescence (IR model) of monocytes and lymphocytes with the residence of the study participants. **g** Scatter plot of predicted senescence (Doxo model) of monocytes and lymphocytes with the body mass index of the study participants. **h** Scatter plot of predicted senescence (Doxo model) of monocytes and lymphocytes with the gender of the study participants. **i** Scatter plot of predicted senescence (Doxo model) of monocytes and lymphocytes with the residence of the study participants. **j** Scatter plot of predicted senescence (Atvr model) of monocytes and lymphocytes with the body mass index of the study participants. **k** Scatter plot of predicted senescence (Atvr model) of monocytes and lymphocytes with the gender of the study participants. **l** Scatter plot of predicted senescence (Atvr model) of monocytes and lymphocytes with the residence of the study participants. **m** Scatter plot of predicted senescence (Anti model) of monocytes and lymphocytes with the body mass index of the study participants. **n** Scatter plot of predicted senescence (Anti model) of monocytes and lymphocytes with the gender of the study participants. **o** Scatter plot of predicted senescence (Anti model) of monocytes and lymphocytes with the residence of the study participants.
